# Supplementary material for: Prognostic Significance and Emerging Predictive Potential of Interleukin-1β Expression in Oncogene-Driven NSCLC
Source: Cancers (Basel). 2025 Sep 3;17(17):2895. doi: 10.3390/cancers17172895 (PMC12428403; doi:10.3390/cancers17172895)
Supplement: Supplementary file 1 [file cancers-17-02895-s001.zip › cancers-3828288-supplementary.pdf]

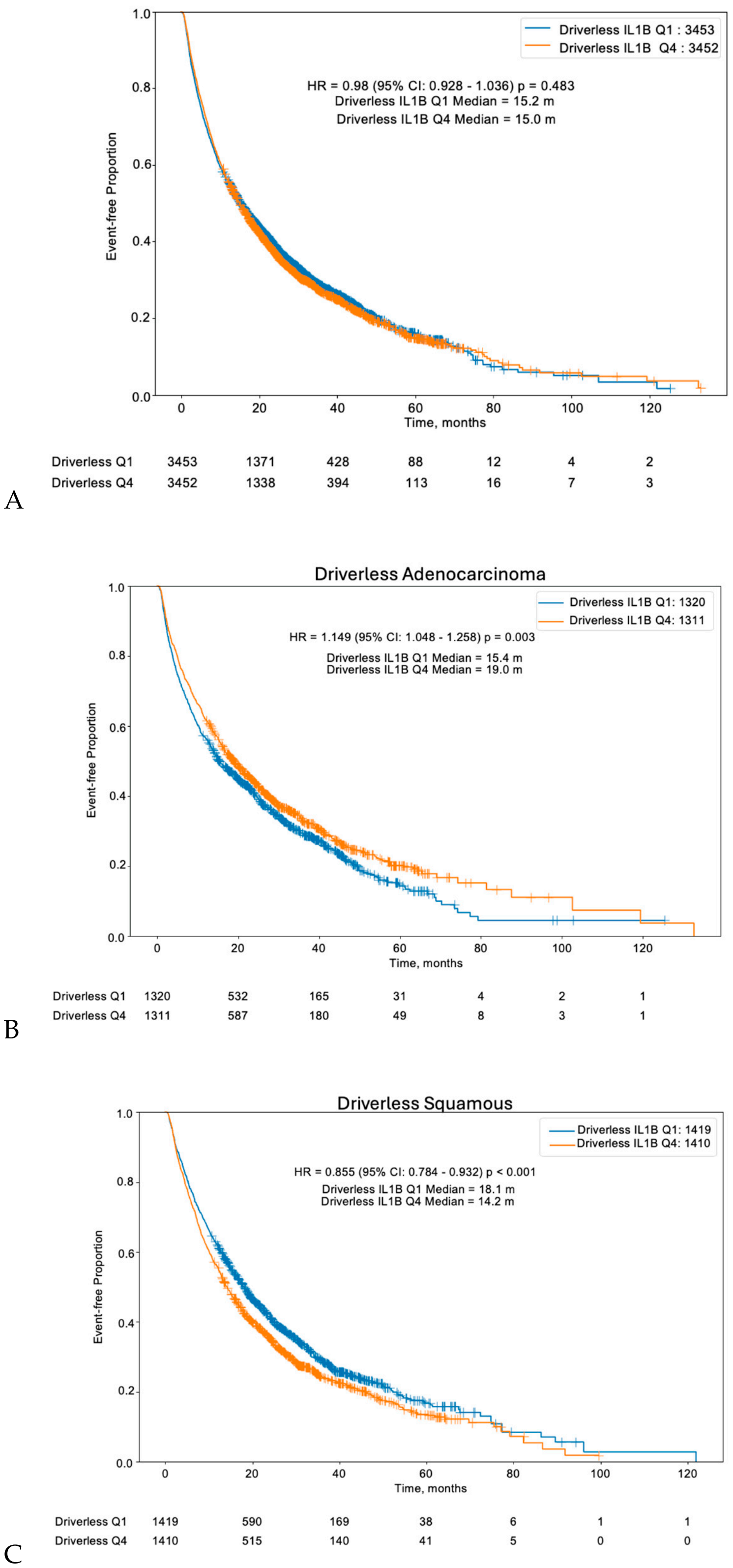

Figure S1. Overall Survival in NSCLC Without Actionable Oncogenic Mutations. Kaplan-Meier curves comparing overall survival (OS) between IL-1β Q1 (low expression) and Q4 (high expression) groups in patients with NSCLC lacking actionable oncogenic mutations. A. All driver-negative NSCLC patients. B. Subset with adenocarcinoma histology. C. Subset with squamous cell carcinoma histology. Hazard ratios (HRs), 95% confidence intervals (CIs), and median OS values are shown. Log-rank *p* values were used to assess statistical significance.

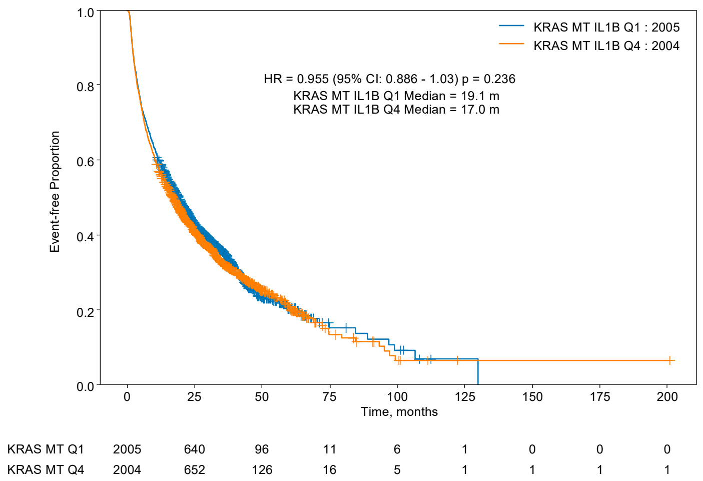
KRAS MT IL1B Q1 : 2005
KRAS MT IL1B Q4 : 2004
HR = 0.955 (95% CI: 0.886 - 1.03) p = 0.236
KRAS MT IL1B Q1 Median = 19.1 m
KRAS MT IL1B Q4 Median = 17.0 m
Event-free Proportion
Time, months
KRAS MT Q1 2005 640 96 11 6 1 0 0 0
KRAS MT Q4 2004 652 126 16 5 1 1 1 1

A

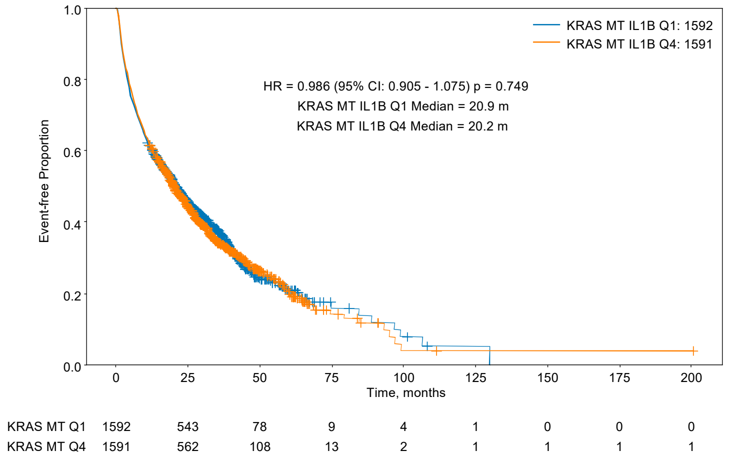
KRAS MT IL1B Q1: 1592
KRAS MT IL1B Q4: 1591
HR = 0.986 (95% CI: 0.905 - 1.075) p = 0.749
KRAS MT IL1B Q1 Median = 20.9 m
KRAS MT IL1B Q4 Median = 20.2 m
Event-free Proportion
Time, months
KRAS MT Q1 1592 543 78 9 4 1 0 0 0
KRAS MT Q4 1591 562 108 13 2 1 1 1 1

B

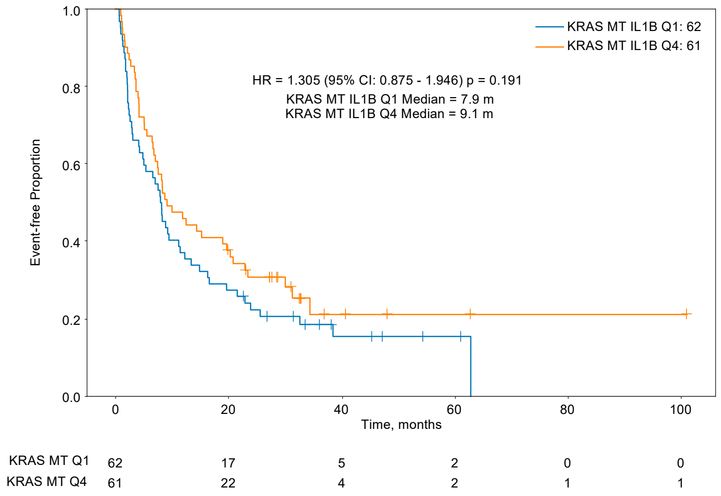
KRAS MT IL1B Q1: 62
KRAS MT IL1B Q4: 61
HR = 1.305 (95% CI: 0.875 - 1.946) p = 0.191
KRAS MT IL1B Q1 Median = 7.9 m
KRAS MT IL1B Q4 Median = 9.1 m
Event-free Proportion
Time, months
KRAS MT Q1 62 17 5 2 0 0
KRAS MT Q4 61 22 4 2 1 1

C

Figure S2. Overall Survival in KRAS-Mutated NSCLC. Kaplan-Meier curves comparing overall survival (OS) between IL-1β Q1 (low expression) and Q4 (high expression) groups in patients with KRAS-mutated non–small cell lung cancer (NSCLC). A. All patients with KRAS-mutated NSCLC. B. Patients with KRAS-mutated adenocarcinoma. C. Patients with KRAS-mutated squamous cell carcinoma. Hazard ratios (HRs), 95% confidence intervals (CIs), and median OS in months are shown. Log-rank *p* values assess survival differences between IL-1β expression groups.

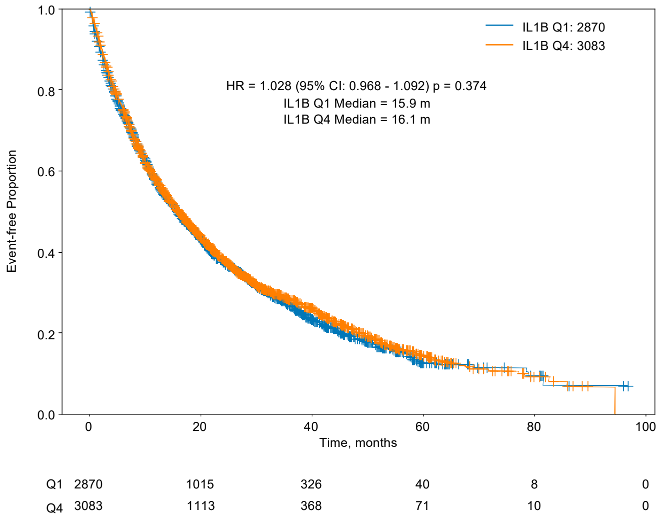

A

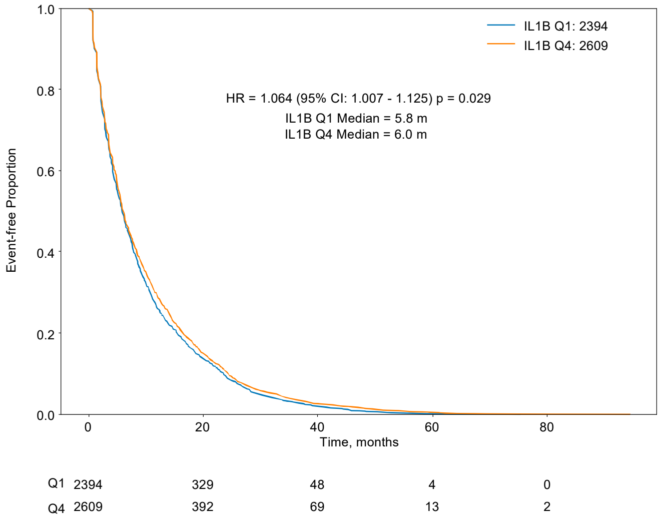

B

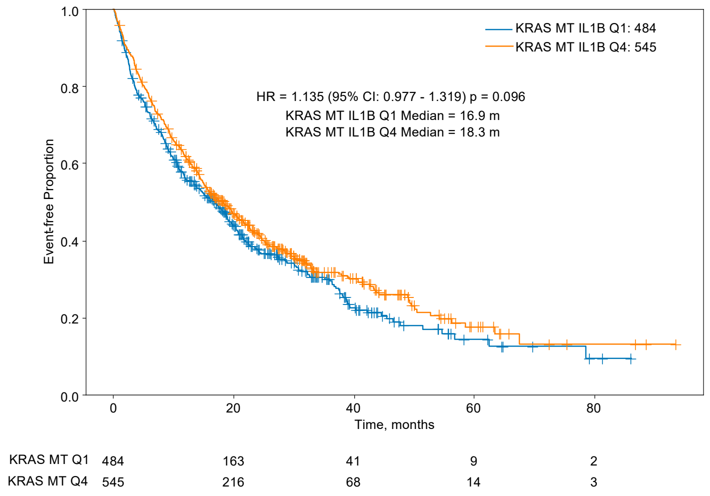

C

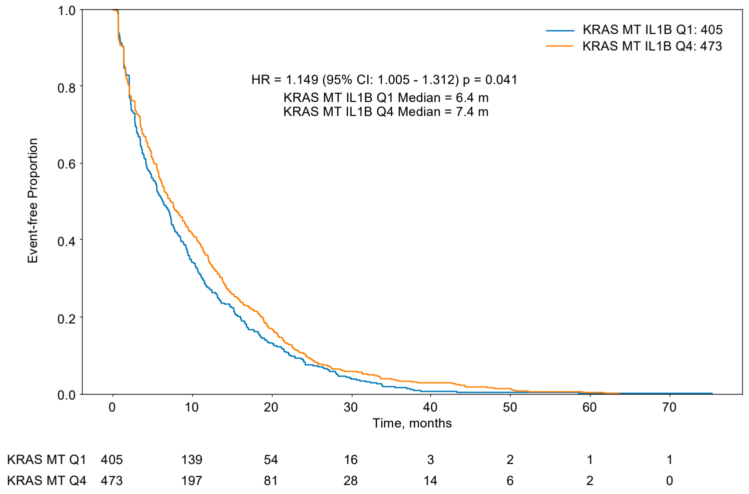

D

Figure S3. Overall Survival and Time on Treatment (TOT) in NSCLC Treated With Pembrolizumab. Kaplan-Meier curves comparing IL-1β Q1 (low expression) and Q4 (high expression) groups in patients with non–small cell lung cancer (NSCLC) treated with pembrolizumab-based therapy. A. Overall survival (OS) in all patients regardless of histology subtype. B. Time on treatment (TOT) in all patients regardless of histology subtype. C. OS in patients with KRAS-mutated adenocarcinoma. D. TOT in patients with KRAS-mutated adenocarcinoma. Hazard ratios (HRs), 95% confidence intervals (CIs), and median duration in months are indicated on each plot. Log-rank *p* values were used to compare outcomes between IL-1β expression quartiles.
